# Logistics Center Selection and Logistics Network Construction from the Perspective of Urban Geographic Information Fusion

**DOI:** 10.3390/s24061878

**Published:** 2024-03-14

**Authors:** Zhanxin Ma, Xiyu Zheng, Hejun Liang, Ping Luo

**Affiliations:** 1 China Institute of FTZ Supply Chain, Shanghai Maritime University, Shanghai 201306, China; mazx@shmtu.edu.cn (Z.M.); 202330210356@stu.shmtu.edu.cn (X.Z.); 2 College of Engineering Science and Technology, Shanghai Ocean University, Shanghai 201306, China; hjliang@shou.edu.cn; 3 Department of Statistics, Shanghai University of Finance and Economics Zhejiang College, Jinhua 321013, China

**Keywords:** last mile, logistics nodes, clustering analysis, greedy algorithm

## Abstract

The last-mile logistics in cities have become an indispensable part of the urban logistics system. This study aims to explore the effective selection of last-mile logistics nodes to enhance the efficiency of logistics distribution, strengthen the image of corporate distribution, further reduce corporate operating costs, and alleviate urban traffic congestion. This paper proposes a clustering-based approach to identify urban logistics nodes from the perspective of geographic information fusion. This method comprehensively considers several key indicators, including the coverage, balance, and urban traffic conditions of logistics distribution. Additionally, we employed a greedy algorithm to identify secondary nodes around primary nodes, thus constructing an effective nodal network. To verify the practicality of this model, we conducted an empirical simulation study using the logistics demand and traffic conditions in the Xianlin District of Nanjing. This research not only identifies the locations of primary and secondary logistics nodes but also provides a new perspective for constructing urban last-mile logistics systems, enriching the academic research related to the construction of logistics nodes. The results of this study are of significant theoretical and practical importance for optimizing urban logistics networks, enhancing logistics efficiency, and promoting the improvement of urban traffic conditions.

## 1. Introduction

Logistics centers are pivotal in the urban logistics framework, evolving from conventional urban freight distribution centers to integral urban area freight hubs due to modern business model transformations. This evolution is critical in bolstering last-mile logistics, thereby enhancing enterprises’ service capabilities and social reputation. Support for freight dispatching from advanced logistics centers to last-mile community logistics centers is crucial. These centers facilitate not only the dispatching of freight across multiple community centers but also coordination among larger centers, which is vital for enhancing the urban logistics service capabilities of enterprises. Moreover, a strategic distribution of logistics centers can significantly improve the efficiency of goods transportation and operational efficiency of logistics firms.

### 1.1. Logistics Center Site Selection

The location of a logistics center is crucial within the logistics system, as it influences the system’s structure, shape, and mode. It affects not only the center’s operational costs, performance, and future development but also the efficiency of the entire logistics system. Thus, choosing the right location for logistics centers is of significant strategic importance for enhancing regional logistics efficiency and optimizing the logistics system structure.

The problem of logistics center location involves selecting one or several logistics distribution centers from multiple logistics nodes to optimize the entire logistics system considering specific objectives and constraints. Proper planning of logistics center locations can enhance the transportation efficiency of the entire logistics network, boosting overall profitability. Improper distribution of logistics center nodes could lead to accumulation of goods at distribution centers, causing delays in the logistics network. Consequently, developing optimization models for the location of distribution centers to address real-world issues remains a key research focus in the logistics field.

In recent years, scholars from both domestic and international arenas have delved deeply into the site selection of logistics centers, proposing a myriad of optimization models and algorithms that are both theoretically sound and practically valuable. The judicious selection of urban logistics center locations is crucial not only for the operational efficiency of logistics activities but also plays a pivotal role in determining logistics costs. Given that logistics costs significantly influence enterprise profits, particular emphasis has been placed on transportation costs within the realm of logistics optimization.

Emre and colleagues [1] combined Geographic Information Systems (GIS) and Binary Particle Swarm Optimization (BPSO) algorithms to offer a holistic solution to urban logistics center site selection. GIS is employed to generate the necessary spatial information for the p-median model, while BPSO is harnessed to ascertain the optimal solution, taking logistics costs into account. Furthermore, Yazdani et al. [2] developed a two-stage decision model, extending the data envelopment analysis (DEA), rough full consistency (R-FUCOM), and combined compromise solution (R-CoCoSo) methods in a rugged environment to identify the most suitable regions for establishing logistics centers across various autonomous communities in Spain. Fernández and colleagues [3] introduced a discrete competitive facility location problem with nonlinear constraints, which includes a minimum market share constraint, and solved it using heuristic algorithms. Heine et al. [4] presented an approximate algorithm for strategic location decision making for logistics centers based on accurate estimates of operational routing costs, which offers verified worst-case guarantees regarding runtime and solution quality.

However, in the urban logistics sector, the necessity of considering multiple logistics center locations arises. Ismail and Fahrettin [5] employed a spatial multi-criteria decision-making method, integrating complex problem structures, expert insights, geographical features, and mathematical modeling to analyze multiple logistics center locations and minimize costs. Jun and associates [6] introduced three socio-economic indicators—economic development, traffic congestion levels, and total logistics demand—and crafted a two-stage model that enhances clustering algorithms and the centroid method, addressing multi-facility issues in practical scenarios. Maryam and Hyunsoo [7] focused on minimizing transportation costs between nodes, utilizing an integrated meta-heuristic algorithm that merges Particle Swarm Optimization (PSO) and Genetic Algorithm (GA) to solve the challenge of optimal site selection for logistics centers. Ge and others [8] explored the facility location problem in the U.S. fresh produce supply chain, proposing a model that incorporates empirical scenarios to acquire vital information for making optimal location decisions. Blanco et al. [9] constructed a general modeling framework for a multi-type maximal covering location problem, introducing a natural nonlinear model, reformulating it into an integer linear programming model, and developing a branch-and-cut algorithm for improved resolution. Yingyi and colleagues [10] enhanced the one-dimensional target constraint location model, proposing a multi-factor constrained P-median model that accounts for operating costs and employing the Particle Swarm Algorithm and Immune Genetic Algorithm to identify an optimal location. Jalal and others [11] proposed a multi-product, multi-period, multi-mode network design and distribution planning decision-making mathematical model that includes the flow of high-value goods, transportation modes, freight types, fleet size, and escort services, introducing a local branching mathematical method that leverages the hierarchical problem structure. Regal and colleagues [12] introduced a method to define zoning procedures based on socio-economic, spatial, and logistical intensity variables, aiding in urban characteristic description and comparison of their outcome profiles, thereby supporting ongoing work on urban features and urban logistics geography.

Nevertheless, with swift economic advancement and evolving customer demands, the initially optimal locations of logistics centers may become less suitable. In response, Liying and others [13] introduced transportation costs between adjacent stages, developing a multi-stage dynamic location model. Concurrently, Juan and colleagues [14] enhanced the Cuckoo Search Algorithm with a balanced learning strategy, while Jeng-Shyang and others [15] introduced an intelligent evolutionary algorithm inspired by the Rafflesia’s living habits—the Rafflesia Optimization Algorithm—to address the conundrum of logistics distribution center site selection.

In the modern urban logistics systems, logistics costs are no longer the sole major factor for consideration. With the promotion of the concept of coordinated development of the natural environment, technology, economy, and society, sustainability has become one of the important goals when selecting the sites of logistics centers. Therefore, traditional methods of constructing logistics centers are insufficient for the development of urban community logistics centers. Rémy and others [16] employed a two-level multi-commodity network flow model to solve the urban center parcel distribution problem and assessed its impact on sustainable development by focusing on carbon emissions. Congjun and others [17], considering sustainability, optimized the site selection of logistics centers based on the 2-tuple linguistic representation model decision method. Moreover, with greenhouse gas emissions as a key factor, Hongzan and others [18] used truck trajectory data combined with DBSCAN clustering and an improved P-median model to determine the best location for urban logistics centers to reduce emissions. As an integral part of urban logistics, cold chain logistics was modeled by Siying and others [19] using a two-level programming approach, and Xinguang and Kang [20] employed a multi-objective location model to solve the site selection problem of cold chain logistics distribution centers considering carbon emissions.

In situations such as natural disasters, the urgency of the context elevates transportation efficiency to a paramount concern. Xenofon and Christos [21] employed a blend of classic heuristic algorithms, forecasting models, and deep neural networks to optimize the selection of distribution center locations. This approach is designed to swiftly expedite the delivery of aid materials to affected areas. Similarly, Kuo-Hao et al. [22] introduced a two-stage stochastic programming model aimed at enhancing the effectiveness of logistics operations for relief materials during critical times. Parragh and colleagues [23] embarked on modeling and solving a bi-objective stochastic facility location problem, grounded in the context of disaster relief and public facility siting. Concurrently, Zengxi et al. [24] integrated Multi-Criteria Decision Making (MCDM) with Geographic Information Systems (GISs) to refine the site selection process for Emergency Logistics Centers (ELCs). This integration aims to hasten the distribution of relief materials, thereby addressing immediate needs during emergencies.

Although current research primarily focuses on reducing costs, minimizing carbon emissions, and improving efficiency through reasonable site selection, the rapid development of urban logistics and the ongoing changes in urban distribution indicators mean that finding optimal solutions among multiple objectives may not be sufficient to meet new challenges. Therefore, this paper proposes the introduction of the concept of multi-data fusion, considering diverse data information, to construct a new logistics planning and design system.

### 1.2. Multi-Data Fusion

Multi-data fusion technology involves integrating datasets from different distributions, sources, and types into a unified global space to form a more consistent expression. This technology occupies an important position in modern information processing and has been widely applied in multiple fields. Compared to the independent processing of a single data source, the advantages of multi-data fusion are significant: it not only improves the detectability and credibility of targets but also broadens the spatio-temporal perception range, reduces the ambiguity of inference, and enhances detection accuracy. Additionally, it increases the dimensional complexity of target features, improves spatial resolution, and enhances the system’s fault tolerance.

In practical applications, multi-sensor systems leverage diverse information sources to garner comprehensive insights about experimental subjects, facilitating real-time monitoring. For instance, Bai et al. [25] employed a multi-data fusion approach that integrates near-infrared spectroscopy with machine vision to evaluate the fermentation level of black tea. Eyob et al. [26] constructed a continuous stream time series using terrestrial and remote sensing data combined with various machine learning algorithms. Their method included eight base models, three meta-learners, and five input scenarios, utilizing a fusion of remote-sensing-based vegetation indices, precipitation, and ground-measured rainfall data to estimate daily flow rates. To tackle the challenge of estimating chlorophyll-a concentration in eutrophic lakes, Cheng et al. [27] introduced a multi-source data fusion method grounded in Bayesian Inference (BIF), effectively harnessing the strengths of in situ observations and remote sensing data. In a similar vein, Yongyun et al. [28] developed a cost-effective, highly accurate biosensor for real-time monitoring of dissolved oxygen in microbial fuel cell biosensors, enhancing its performance through a multi-source data fusion predictive model that incorporates various environmental indicators. Additionally, Hui et al. [29] achieved online monitoring of ethanol’s simultaneous saccharification and fermentation process by integrating a convolutional neural network (CNN) with a recurrent neural network (RNN) in a novel cross-perception multi-source data deep fusion model. Zhang Yi et al. [30] proposed an interactive platform architecture for managing provincial power grid voltage dips using multi-source data fusion, addressing issues like excessive monitoring and limited application. Finally, Qilin et al. [31] introduced a multi-data fusion calibration method for all parameters of the Orbital Multi-View Dynamic Photogrammetry System (OMDPS), enhancing the spatial reference accuracy for spacecraft attitude measurements.

Challenges such as environmental complexity, noise interference, system instability, and diverse algorithmic approaches can impair the precision, completeness, and reliability of extracted feature information during experiments. To surmount these obstacles, Yan et al. [32] devised a collaborative strategy that marries deep learning with machine learning theory to enhance the tracking quality of rice, thereby improving the fusion system’s detection performance. Junyi et al. [33] developed an enhanced real-time target detection system for intelligent vehicles, employing a multi-source fusion method within the ROS Melody software (Melodic) development environment and the NVIDIA Xavier hardware platform (NVIDIA, Santa Clara, CA, USA). To boost the predictive accuracy and efficiency of coal mine gas generation patterns, Huice et al. [34] proposed a method based on multi-source data fusion. Yajie et al. [35] improved soil moisture measurement accuracy by integrating GNSS-IR technology with optical remote sensing, utilizing a multi-data fusion approach based on the Genetic Algorithm–Back-Propagation Neural Network (GAP-NN). In the industrial realm, aiming to minimize unexpected downtimes and extend the lifespan of crucial production machinery, Shreyas et al. [36] applied an interpretable artificial intelligence methodology combined with multi-sensor data fusion, marking a significant advancement in intelligent manufacturing and predictive maintenance within Industry 4.0.

Beyond real-time monitoring, Xueying et al. [37] introduced a multi-source data feature fusion method based on deep learning, addressing the challenge of multi-feature contribution differential analysis in predicting soil carbon content using VNIR and HIS technologies. Jihao et al. [38] crafted a slope estimation algorithm that relies on multi-model and multi-data fusion, enhancing a vehicle’s capability to track real-time road slope changes. In maritime activities, Ye et al. [39] developed an Adaptive Data Fusion (ADF) model using multi-source AIS data to predict ship trajectories effectively. In complex industrial settings like sintering, Yuxuan et al. [40] proposed a quality prediction model that merges industrial camera video data with process parameters. Moreover, Yiqi et al. [41] established a deep learning approach based on multi-data fusion for water quality prediction in urban sewer networks, considering environmental, social, and water quantity indicators, along with monitorable water quality standards. In the context of economic dispatch in power systems, Guanghui and Weixin [42] blended the Particle Swarm Optimization (PSO) and Artificial Fish Swarm Algorithm (AFSA) to introduce a novel hybrid intelligent algorithm, constructing a comprehensive scheduling optimization model under multi-data fusion and multi-objective constraints.

The development of multi-data fusion technology continues to evolve. For instance, Bo and others [43] proposed a Digital Twin Model (DTM) based on Transfer Learning and Multi-Source Data Fusion (DTM-TL-MSDF). This method effectively integrates experimental and simulation data, aiming to construct an accurate digital twin model for real-time monitoring of structural strength. Sizhe and others [44] developed a novel GeoAI research method that performs deep machine learning from multi-source geospatial data to effectively detect natural features. Moreover, Nan and others [45] proposed a new architecture for a Trusted Execution Environment with integrated blockchain capabilities, aiming to improve the efficiency of multi-source data fusion processing under the constraints of business scenarios.

In recent years, although research on multi-data fusion has mainly focused on data monitoring or prediction under dynamic changes, there are relatively few discussions on the location of logistics centers. However, in the context of the rapid development of urban logistics industry, a single factor is no longer sufficient to meet the demand for the optimality of logistics center location. Therefore, in the complex environment of solutions to the urban logistics center location analysis process, in order to make a reasonable logistics center location decision, it is necessary to evaluate different decision making factors, especially geographic information data. Because geographic information is a technical means of acquiring, processing, and analyzing spatial data, it can be used to collect, analyze, and apply data on the location of logistics centers. Further, this paper adopts a multidimensional data fusion approach from the perspective of geographic information fusion in the study of logistics center locations, focusing on geographically relevant indicators around the logistics nodes, such as logistics and distribution coverage, equilibrium, and urban congestion, in order to construct a more accurate logistics location model.

Moreover, when constructing logistics nodes, this paper also gives special consideration to transportation fluency. To this end, five selection objectives are determined: low operation rate, low rate of change of traffic congestion, high coverage rate of nodes at 3 km, short distance between logistics parks and the nearest city-level nodes, and high efficiency of cargo transportation. Meanwhile, from the perspective of the overall logistics system, focusing on the balance and rationality of the system’s operation, the decentralization of primary nodes and the aggregation of secondary nodes are established as the selection objectives. This study proceeds to establish a multi-objective node selection model and applies cluster analysis to simply cluster the entire region into small regions of similar size. Within each small region, a clustering center, i.e., the initially identified primary node, is identified. Then, the clustering center is dynamically adjusted by the K-mean algorithm, and the distance, i.e., the similarity, between the other nodes within each small region and the initially determined first-level node is calculated. The clustering results are optimized through repeated iterations to minimize the sum of squares of the distances of all categories to their respective category centers, thus determining the final first-level nodes and their jurisdictional areas within 3 km. Compared with other models, this model not only obtains the optimal solution faster, but also performs better in balancing the optimal distribution and coverage of logistics centers, which leads to a more reasonable site selection scheme.

## 2. Modeling Multi-Objective Node Selection

To facilitate understanding and model construction, we have defined the parameters of the model as follows.

Let the total number of nodes be N, and the coordinates of each node are represented as $\left(X_{i},Y_{i}\right)$, where i=1,2……N. Define the coordinates of primary node $F_{i}$ as $\left(X_{F_{i}},Y_{F_{i}}\right)$, the coordinates of secondary node $S_{i}$ as $\left(X_{S_{i}},Y_{S_{i}}\right)$, and the coordinates of the logistics park $W_{i}$ as $\left(X_{W_{i}},Y_{W_{i}}\right)$. Assume there are $W_{k}$ logistics parks, where k=1,2,3,4; and each logistics park $W_{k}$ corresponds to a primary node $F_{W_{k}}$.

Let the total number of primary nodes be NF, and define the set of secondary nodes belonging to primary node $F_{i}$ as

$${SF}_{i}=\left\{S_{i1},S_{i2},S_{i3},\ldots \ldots S_{iNF_{i}}\right\},$$

where ${NF}_{i}$ represents the number of secondary nodes contained in primary node $F_{i}$. Assuming the total number of secondary nodes is NE, then $NE=\sum_{i=1}^{NF}{NF}_{i}$.

Let $A_{ij}$ be the total freight in and out volume between nodes i and j; let $I_{ij}$ be the inbound freight volume from the area corresponding to node i to the area corresponding to node j; let ${AH}_{i}$ be the actual total ground freight in and out volume of node i before the establishment of the underground logistics system, and ${YH}_{i}$ be the actual total ground freight in and out volume of the node after the establishment of the underground logistics system.

Finally, define the set ${GT}_{i}$ as the set of nodes covered by node i within a 3 km range:

$${GT}_{i}=\left\{G_{i1},G_{i2},G_{i3},\ldots \ldots G_{iT_{i}}\right\},$$

where $T_{i}$ is the number of elements in the set ${GT}_{i}$.

### 2.1. Multi-Objective Node Selection Model

The primary goal of developing urban underground logistics networks is to alleviate or even eliminate traffic congestion, achieving at least basic traffic flow. Starting from this main goal, we analyzed various specific factors that might affect urban traffic and established the following five main selection objectives: low turnover rate, low traffic congestion change rate, high node 3 km coverage rate, short distance between logistics parks and the nearest primary node, and high goods transportation efficiency. Additionally, considering the overall logistics system’s balance and rationality, we also determined the dispersion of primary nodes and the aggregation of secondary nodes as selection objectives.

Objective 1: Minimize Turnover Rate

We defined the total transport volume from logistics park $W_{k}$ to its corresponding primary node $F_{W_{k}}$ as ${IT}_{k}$, and the total transport volume from primary node $F_{W_{k}}$ to other primary nodes as ${OT}_{k}$. Therefore, the turnover rate of primary node $F_{W_{k}}$, i.e., the ratio of total output to total input of goods, is defined as:

$$\min{RT}_{k}=\frac{{OT}_{k}}{IT_{k}}$$

Objective 2: Maximize Node 3 km Coverage Rate

We define ${GT}_{i}$ as the set of nodes covered by node i within a 3 km range, and $\#\left\{\overset{N}{\underset{i=1}{⋃}}{GT}_{i}\right\}$ represents the number of nodes covered by node i within a 3 km range. Thus, the 3 km coverage rate of node i, i.e., the ratio of the number of covered nodes to the total number of nodes, is defined as

$$\max RFG=\frac{\#\left\{\overset{N}{\underset{i=1}{⋃}}{GT}_{i}\right\}}{N},$$

where #{ } represents the number of elements in the set.

Objective 3: Minimize Traffic Congestion Change Rate

We define f as the functional relationship between logistics volume and traffic congestion. $f\left({AH}_{i}\right)-f\left({YH}_{i}\right)$ represents the change in ground traffic congestion before and after the construction of the underground logistics system. Therefore, the traffic congestion change rate is defined as

$${RJ}_{i}=\frac{f\left({AH}_{i}\right)-f\left({YH}_{i}\right)}{f\left({AH}_{i}\right)}\times 100\%$$

$$\min RJ=\frac{\sum_{i=1}^{N}{RJ}_{i}}{N}$$

Objective 4: Minimize Distance Between Logistics Park and Nearest Primary Node

We define ${WS}_{i}=\left\{W_{i1},W_{i2},\ldots \ldots W_{i{WN}_{i}}\right\}$ as the set of primary node numbers corresponding to logistics park ${WS}_{i}$, with a count of ${WN}_{i}$. Thus, the distance between the logistics park and the nearest primary node is defined as

$$\min WFD=\frac{\sum_{i=1}^{4}\sum_{j\in {WS}_{i}}\sqrt{{\left(X_{W_{i}}-X_{j}\right)}^{2}+{\left(Y_{W_{i}}-Y_{j}\right)}^{2}}}{\sum_{i=1}^{4}{WN}_{i}}$$

Objective 5: Maximize Goods Transportation Efficiency

Considering the stability of the underground logistics system operation, maximizing goods transportation efficiency is crucial. Transportation efficiency can be measured by the ratio of goods transport volume to the total transportation time (including waiting time at nodes and time spent in transit). Hence, we define Objective 5 as follows

$$\max{TR}_{ij}=\frac{NJ\times M}{{NNF}_{ij}\times 12+\frac{D_{ij}}{V}}$$

where ${TR}_{ij}$ represents the maximum volume of goods transported from node i to node j per unit of time; ${NNF}_{ij}$ is the number of primary nodes passed during the transport of goods from node i to node j; V is the speed of shuttle transportation; NJ is the maximum number of vehicles per shuttle; $D_{ij}$ is the total distance from node i to node j; and M is the volume of goods transported per shuttle, for example, 5 tons or 10 tons.

Objective 6: Maximize Dispersion of Primary Nodes

Let $\left(X_{F},Y_{F}\right)$ be the central point of all primary nodes. The dispersion of primary nodes can be measured by the standard deviation of the distances from each primary node to the central point of primary nodes, defined as follows:

$$\max CF=\sqrt{\sum_{k=1}^{NF}{\left(X_{F_{k}}-\bar{X_{F}}\right)}^{2}+{\left(Y_{F_{k}}-\bar{Y_{F}}\right)}^{2}}$$

Objective 7: Maximize Aggregation of Secondary Nodes

To enhance the transportation efficiency among secondary nodes covered by each regional primary node, we use the aggregation of secondary nodes to measure this efficiency. The aggregation of secondary nodes is defined as the standard deviation of the distances between each primary node and its covered secondary nodes, as follows:

$$\max CCE=\sum_{k=1}^{NF}\sum_{S_{i}\in {SF}_{i}}\sqrt{{\left(X_{F_{k}}-X_{S_{i}}\right)}^{2}+{\left(Y_{F_{k}}-Y_{S_{i}}\right)}^{2}}$$

### 2.2. Restrictive Condition

Constraint One: Ensure Underground Goods Transportation Meets Actual Demand

To ensure the underground logistics system can meet actual freight demands, we set a constraint that the underground goods transportation volume at each node must be greater than a certain percentage θ of the actual freight demand, while also limiting the total amount of goods entering and exiting each node on the ground. The specific expression is as follows:

$$\sum_{i=1}^{{NF}_{i}}{IO}_{i}\geq \left(\sum_{j=1}^{NE}\sum_{i=1}^{NF}A_{ij}\right)\times \theta$$

$${IO}_{i}\leq {IO}_{max}\left\{\begin{array}{c} \mathrm{when}\;i\;\mathrm{is}\;a\;\mathrm{primary}\;\mathrm{node},\;{IO}_{max}=4000t\\ \mathrm{when}\;i\;\mathrm{is}\;a\;\mathrm{secondary}\;\mathrm{node},{IO}_{max}=3000t \end{array}\right.$$

Constraint Two: Promote Traffic Flow

To alleviate traffic congestion and ensure basic road traffic flow, the construction of the underground logistics system needs to satisfy the following constraint:

$$f\left({YH}_{i}\right)\leq 4$$

$$f=k\times {YH}_{i}$$

Constraint Three: Control Ground Goods Volume Change

The difference in ground goods volume ${YH}_{i}$ before and after the construction of the underground logistics system must be controlled within a certain range ζ to maintain the overall stability of the logistics system:

$${YH}_{i}=\left\{\begin{array}{c} {AH}_{i}-{MT}_{i},\mathrm{when}\;{AH}_{i}\geq {MT}_{i}\\ \;0,\;\mathrm{when}\;{AH}_{i}<{MT}_{i} \end{array}\right.$$

$${YH}_{i}\leq \zeta$$

Dimensionless treatment: To facilitate solving the multi-objective programming problem, differently dimensioned objective functions are normalized. For the objective $f_{i}$, if smaller is better, we find the minimum (best) and maximum (worst) values among n choices; if larger is better, vice versa. The dimensionless value can be obtained by linear interpolation, i.e.,

$$\frac{f_{x}-f_{min}}{f_{max}-f_{min}}$$

In summary, we established a multi-objective programming node selection model:

$$\max{RFG}^{\prime }+{{TR}_{ij}}^{\prime }+{CF}^{\prime }+{CCE}^{\prime }-{{RT}_{k}}^{\prime }-{RJ}^{\prime }-{WFD}^{\prime }$$

$$s.t.\left\{\begin{array}{c} \sum_{i=1}^{{NF}_{i}}{IO}_{i}\geq \left(\sum_{j=1}^{NE}\sum_{i=1}^{NF}A_{ij}\right)\times \theta \\ f\left({YH}_{i}\right)\leq 4\\ f=k\times {YH}_{i}\\ {{YH}_{i}\leq \zeta ,\;YH}_{i}=\left\{\begin{array}{c} {AH}_{i}-{MT}_{i},\mathrm{when}\;{AH}_{i}\geq {MT}_{i}\\ \;0,\;\mathrm{when}\;{AH}_{i}<{MT}_{i} \end{array}\right.\\ {IO}_{i}\leq {IO}_{max}\left\{\begin{array}{c} \mathrm{when}\;i\;\mathrm{is}\;a\;\mathrm{primary}\;\mathrm{node},\;{IO}_{max}=4000t\\ \mathrm{when}\;i\;\mathrm{is}\;a\;\mathrm{secondary}\;\mathrm{node},{IO}_{max}=3000t \end{array}\right. \end{array}\right.$$

## 3. Preferred First-Level Node Identification Based on Cluster Analysis

### 3.1. Cluster Analysis Algorithms

A cluster analysis algorithm is a learning process that aims to identify clustering characteristics within a dataset. Based on clustering criteria, namely threshold criteria and function criteria, the algorithm groups the nodes of each region into a major category by analyzing the similarity between different categories and the threshold of their similarity measure. It further selects primary nodes for each region.

The threshold criterion is a criterion for classification based on a distance threshold. Based on past practical experience, we have defined a similarity measure threshold and used the nearest neighbor rule to determine if certain pattern samples belong to a specific cluster category.

In cluster analysis, the function used to represent the similarity or dissimilarity between patterns is known as the cluster criterion function. This function is a function of the pattern sample set $\left\{X\right\}$ and pattern categories $\left\{S_{j},j=1,2,L,m\right\}$, where m is the number of categories. A common function criterion is the sum of squared errors, also known as the minimum variance partition function, defined as follows:

$$J=\sum_{j=1}^{m}\sum_{X\in S_{i}}{\left\|X-M_{j}\right\|}^{2}$$

where m represents the number of pattern categories, $M_{j}=\frac{1}{N_{j}}\sum_{X=S_{i}}X$ is the mean vector of the sample set $S_{j}$, and $N_{j}$ is the number of samples in $S_{j}$. When the value of J reaches a minimum, it indicates that the classification result is satisfactory; thus, the minimal value of J can be used as the objective function.

In the process of cluster analysis, nodes in different regions show dynamic changes as the clustering becomes progressively refined. To accurately classify nodes in different regions, one of the dynamic clustering methods, the K-means algorithm, is used. This algorithm uses the sum of squared errors as the clustering criterion and iteratively optimizes the clustering results, minimizing the sum of squared distances of all samples to the centers of their respective categories, thus maximizing similarity and enabling samples to be classified into the same category.

The main steps of the K-means algorithm are as follows:

**Step 1:** Initialization: Randomly select K initial clustering centers.

**Step 2:** Calculate distance: Calculate the distance between samples and each clustering center, and allocate samples based on the principle of minimum distance.

**Step 3:** Assignment: Find the nearest clustering center for each sample and calculate the new clustering center.

**Step 4:** Revise clustering center: Recalculate cluster centers based on newly assigned samples.

**Step 5:** Compute deviation: Calculate clustering deviation.

**Step 6:** Convergence judgment: If the clustering centers no longer change, the algorithm terminates; otherwise, return to Step 2.

**Figure 1:**
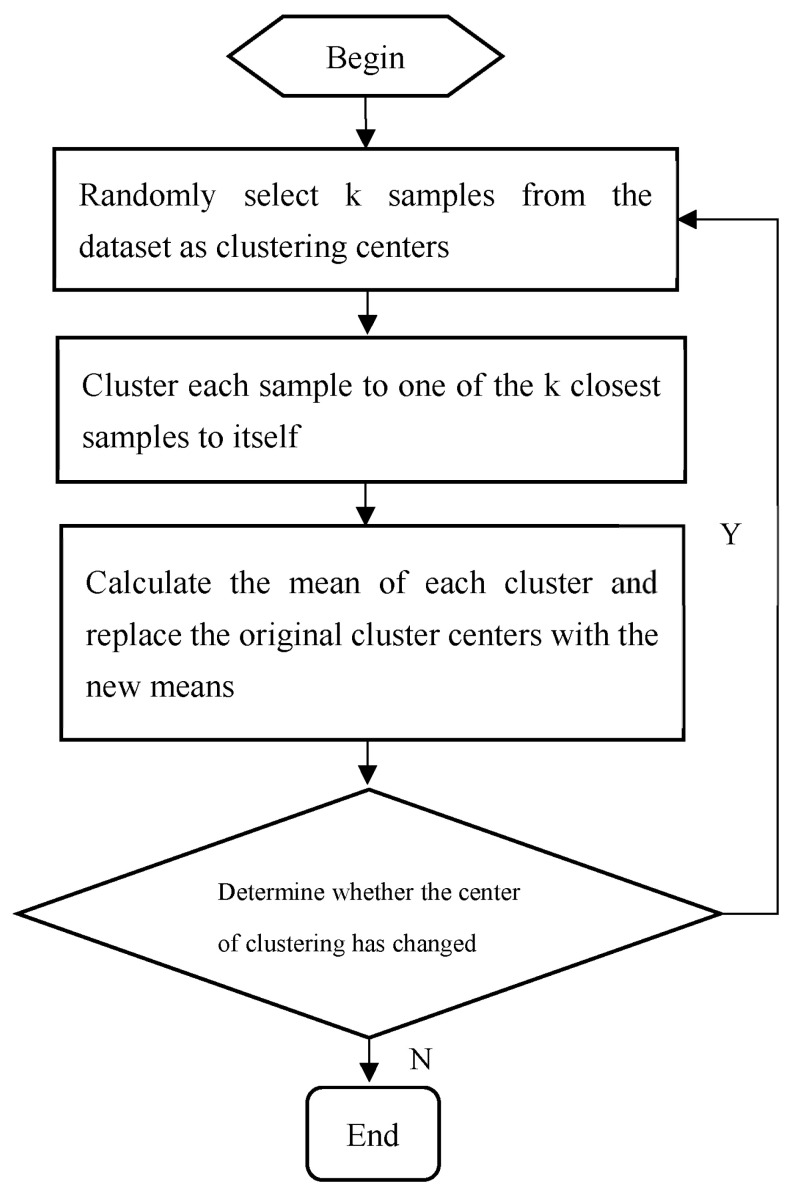
Algorithmic flowchart for dynamic clustering method using K-means.

For the flowchart of the K-means algorithm, see Figure 1.

Given the complexity of the freight area division and the corresponding freight Origin–Destination (OD) flow matrix in the Xianlin area of Nanjing City, we undertook a series of steps to conduct cluster analysis. Initially, based on the freight volume and distance data of various regional nodes, the entire area was simply clustered into small regions of similar size. Next, a clustering center was determined in each small region, associated with other nodes in the region and serving as the preliminarily determined primary node. Then, the distances, i.e., similarities, between other nodes within each small region and the preliminarily determined primary nodes were calculated, ensuring that the similarity was greater than or equal to the set similarity measure threshold. Subsequently, the deviation of the cluster centers was calculated and corrected to determine the cluster center, which is the primary node. Finally, by iteratively optimizing the clustering results, the sum of squared distances of all categories to their respective category centers was minimized, achieving maximum similarity and classifying them into the same category. Through this dynamic clustering process, we determined the primary nodes and their 3 km jurisdiction areas.

### 3.2. Cluster Analysis Results Test

After clustering the regional nodes using the k-means clustering algorithm, we calculated the sum of average distances of each node to the cluster center in each category and normalized it. If this sum of average distances fell within the set mean threshold range, the clustering result could be considered accurate. For different regional nodes, we defined their mean as

$$\bar{n}=\sum_{i=1}^{N}n_{i}$$

and performed the following normalization:

ni~=ni−n¯∑i=1Nni−nN

The center value of each preliminarily classified category i is calculated as follows:

$$C_{j}=\sum_{i=1}^{N_{j}}n_{ij}\;\;\;\;\;\;\;\;j=1,2,\ldots \ldots ,M_{j}$$

where $n_{i}$ represents the distance from the nodes within the i area to the clustering center, N is the total number of nodes in the entire region, $N_{j}$ is the total number of nodes in the i area, and $M_{j}$ is the total number of nodes in the j area. The average distance from different categories to their respective cluster centers is defined as

$$\bar{D}=\frac{\sum_{i=1}^{N_{j}}\sum_{j=1}^{M_{j}}{\left(\tilde{n_{ij}}-C_{j}\right)}^{2}}{N}$$

## 4. Selection of Secondary Node Identification Based on a Greedy Algorithm

To select secondary nodes for underground logistics areas, we employed a greedy algorithm. This section outlines the key characteristics of the greedy algorithm and the specific process for selecting secondary nodes, with the implementation demonstrated using MATLAB (R2018b).

From the perspective of graph theory, an underground logistics area can be abstracted as a graph G=(V, E), where $V_{i}$ represents a connecting node, and $E_{ij}=\left[V_{i},V_{j}\right]$ represents the edge connecting nodes $V_{i}$ and $V_{j}$. Each edge is assigned a non-negative weight $Q\left(E_{ij}\right)=Q_{ij}$, which is determined by both the freight volume and distance between the two nodes. Thus, the process of determining secondary nodes can be viewed as solving the minimum spanning tree problem.

**Figure 2:**
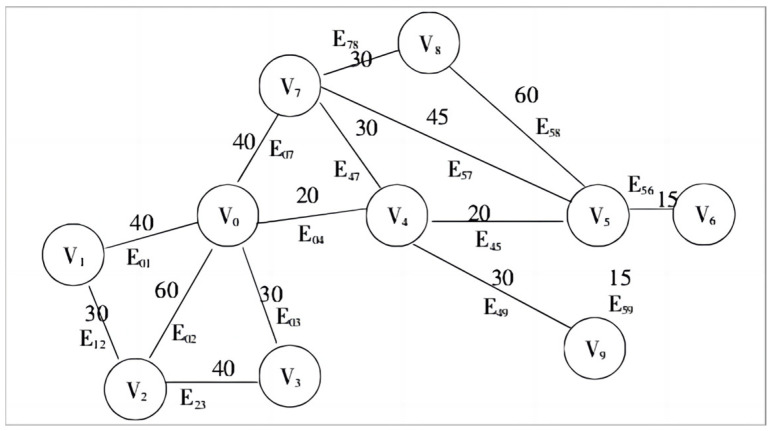
Undirected connectivity map.

As shown in Figure 2, suppose an undirected graph G represents a logistics network, where $V_{0},V_{1},V_{2},V_{3}\ldots \ldots V_{9}$ represent 10 connecting nodes, and $E_{01},E_{02},E_{25}$, etc., represent paths between nodes.

The Dijkstra algorithm was invented by the renowned Dutch computer scientist Edsger W. Dijkstra in the mid-1950s. This algorithm is used to solve the single-source shortest path problem, i.e., finding a series of shortest paths from a given starting point to all other vertices in a weighted connected graph. Unlike other algorithms, Dijkstra’s algorithm does not require visiting the single shortest path of all vertices. Instead, it generates a set of paths from the starting point to different vertices in the graph, some of which may share common edges.

The specific process of the Dijkstra algorithm is as follows:

**Step 1**: Find the weight of the shortest edge between the starting point and the nearest vertex to that starting point.

**Step 2**: Continue to find the second nearest edge. Before the i iteration, the algorithm has already determined i − 1 shortest paths connecting the starting point and its nearest vertices.

**Step 3**: Determine the last required node and stop when all freight volume demands within the region are met.

**Figure 3:**
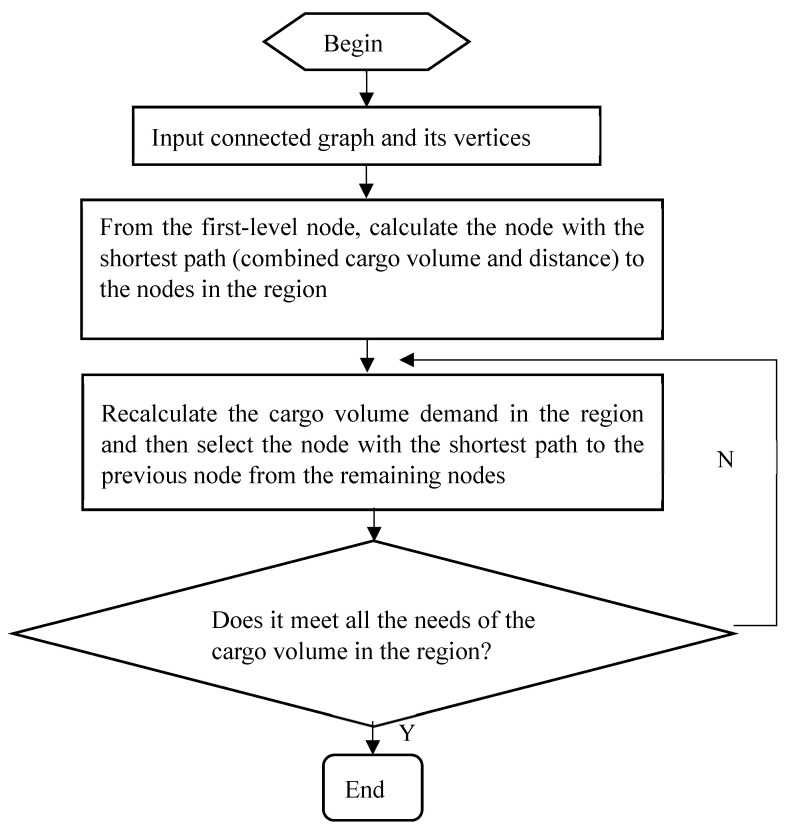
Flowchart of the greedy algorithm for selecting secondary nodes.

After determining primary nodes in Nanjing’s Xianlin district freight area through cluster analysis, we applied the greedy algorithm for secondary node selection. Initially, we modeled the entire area as an undirected graph, dividing the problem into smaller sub-problems. Starting from the primary nodes, we calculated the shortest node and its path within the region based on cargo volume and distance among various regional nodes, identifying these as preliminary secondary nodes. Subsequently, we searched for the next closest node within the area, recalculating the cargo demand and selecting the nearest unchosen node. Through iterative processes, we identified all required nodes, concluding the computation once all regional cargo demands were met. This greedy algorithmic process allowed us to delineate the jurisdiction of all secondary nodes within the primary node areas. See Figure 3.

## 5. Example and Result Analysis

### 5.1. Selection of Underground Logistics Nodes

**Figure 4:**
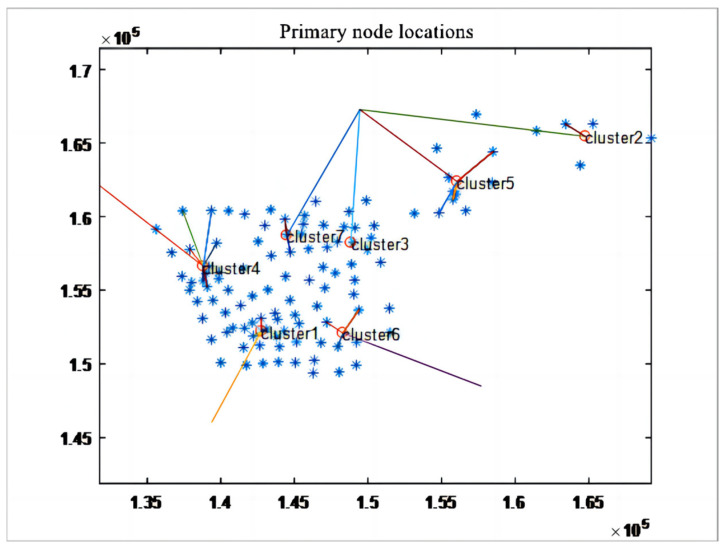
Node location distribution schematic.

By employing a dynamic cluster analysis algorithm, we conducted a detailed analysis of the transportation freight area in Nanjing’s Xianlin district. This method enabled us to identify the coordinates and jurisdiction areas of seven primary nodes (see Figure 4), along with their respective freight volumes. We also calculated the transshipment rates of these primary nodes using MATLAB programming based on the freight Origin–Destination (OD) flow matrix of Xianlin district (see Table 1). Further, the greedy algorithm helped us pinpoint the coordinates of 55 secondary node clusters associated with these primary nodes, including adjacent service nodes and their freight volumes. We analyzed the transshipment activities of secondary nodes and calculated their transshipment rates through MATLAB programming (see Table 2).

### 5.2. Scope of Nodal Services

**Figure 5:**
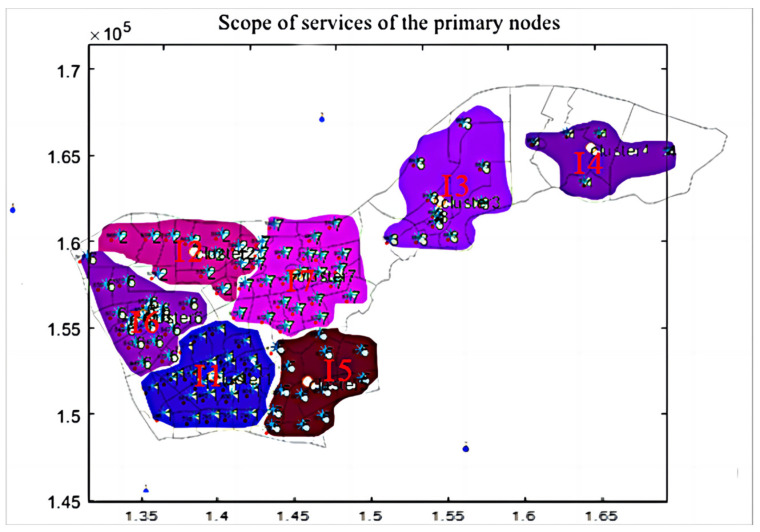
Scope of services map of the seven tier-1 nodes.

To visually represent the service range of each node, we analyzed areas within a 3 km radius centered on each node. Considering the total freight volume and transshipment conditions, we ran a MATLAB program to ascertain the service range of each primary node and their corresponding secondary nodes, including the central points of their service areas (see Table 3). Subsequently, we graphically depicted the actual service areas of the nodes (see Figure 5), thereby clearly illustrating their operational status.

### 5.3. Analysis of Results

The Xianlin district in Nanjing City, serving as a sub-center of the city, includes the Xianhe area, which is a hub of higher education. This region features a combination of multiple universities and residential areas, forming a comprehensive community. Driven by the university industry chain, various small logistics industries have developed rapidly. According to the freight OD data, the typical daily freight volume reached 327,000 tons. Therefore, 15 primary nodes were set up around the Xianlin University Town to accelerate the efficiency of freight transportation.

In the Bai Xiang area of the Xianlin district, an important science and technology industrial park in Nanjing, the development of economic and high-tech industrial parks has led to a relatively high demand for logistics and freight. We established logistics nodes at key transportation hubs and commercial areas such as the Presidential Palace, Jianye Wanda Plaza (Node number 857), and Andemen (Node number 807), to meet the logistics network needs of the region.

The entire logistics network service range reached 289.50 square km, while the total area of Xianlin district is 308.29 square km, achieving a coverage rate as high as 93.91%. This coverage rate satisfies the freight needs of the overall logistics network, enabling the highest turnover rate to reach 0.949.

### 5.4. Results Discussion

In this section, we compare the method introduced in this paper with the multi-objective location and channel model for the ULS network proposed by Hejun et al. [46]. Both approaches plan logistics networks based on actual data such as geographical location, population density, and freight traffic conditions, and both achieved desirable outcomes in alleviating traffic congestion and reducing logistics costs during a logistics network simulation in Nanjing’s Xianlin district. However, our study distinguishes itself by integrating multi-dimensional data such as logistics distribution coverage, balance, and urban congestion from a geographic information fusion perspective, thereby achieving multi-objective optimization and rationalizing the selection of logistics center locations.

The model constructed in this paper has several advantages: First, it builds a robust and effective mathematical model by conducting a thorough analysis of the problem and selecting key variables based on actual data. Second, the model facilitates the identification of urban logistics network centers and their interconnections, making it readily adaptable for rational decision making regarding logistics centers in other urban contexts. Third, the integration of multi-dimensional data offers a superior approach, enabling a more comprehensive consideration of decision making factors in the location selection process for logistics centers. However, there are limitations as well. The identification of urban logistics center nodes relies on cluster analysis and the greedy algorithm, but there are various other methods that could potentially identify these nodes. Additionally, while the model provides acceptable solutions for logistics center location problems in the short term, its effectiveness remains uncertain for larger-scale problems.

## 6. Conclusions

In this paper, against the background of logistics operation enterprises, we study how enterprises can select logistics nodes according to the freight demand and urban traffic conditions in each region from the perspective of geographic information integration. We comprehensively consider the key indicators of geographic information around logistics centers with the aim of meeting the business needs of enterprises. These include operation rate, traffic congestion change rate, nodes’ 3 km coverage, distance between logistics parks and the nearest first-level nodes, cargo transportation efficiency, dispersion of first-level nodes, and clustering of second-level nodes; we take them as the objectives of node selection. In this study, the cluster analysis method was used to construct a first-level node clustering identification model, and the optimization objective and greedy algorithm were applied to identify and analyze the second-level nodes according to the characteristics of the first-level nodes. Finally, by collecting the freight traffic data of Xianlin District in Nanjing and carefully dividing and analyzing its traffic and freight coverage area, we implemented a simulation analysis. The analysis results show that the proposed model is able to cover most of the logistics network service area in Xianlin District, Nanjing and meet the freight transportation demand of the overall logistics network.

From a research perspective, the model developed in this paper has a broader scope compared to existing studies. Previous research has typically concentrated on finding the optimal solution to achieve overarching objectives, such as minimizing logistics costs, maximizing transport efficiency, and reducing carbon emissions. However, these studies often show limited consideration of factors. In contrast, this paper not only addresses these objectives but also introduces a multi-dimensional data fusion approach from the perspective of geographic information fusion. It comprehensively analyzes various indicators such as logistics distribution coverage, equilibrium, and urban congestion, and integrates complex problem structures, geographic features, and mathematical modeling techniques. This approach facilitates multi-objective optimization in urban logistics center siting analyses and establishes an innovative logistics planning and design system, thereby distinguishing this work from the existing literature by offering novel insights into the field of logistics center siting.

From a data collection standpoint, the model underscores the importance and value of utilizing multiple data sources, whether open or proprietary, as decision making factors for logistics center location. Models considering fewer factors may provide a general overview of urban logistics network construction, whereas those incorporating multi-dimensional data fusion offer a more precise logistics planning and design system and insights into urban dynamics.

Practically, the proposed method is applicable to any city with relevant data sources. Indeed, this approach is not only widely applicable but also facilitates inter-city comparisons. Additionally, the inclusion of locale-specific geographic data allows for comparisons between cities, enhancing comparability. Thus, this method supports the siting of logistics centers based on urban geographic characteristics, providing a robust framework for decision making in diverse urban contexts.

Future research will focus on combining multi-data fusion with the construction of a logistics center site selection model in order to further enhance the accuracy of site selection. To this end, we will not only consider enterprise-related factors and urban traffic conditions but also include diverse indicators, such as the residential situation around logistics nodes, in the model.

## Figures and Tables

**Table 1:** Table of information related to the seven first-level nodes.

Level 1 Node Number	Freight Volume/t	Transit Rate	Position Coordinate	
			X	Y
I-1	44,549.12	0.1949	142,746.9	152,213.7
I-2	13,254.41	0.189	164,769.5	165,451.4
I-3	31,508.57	0.1888	148,832.7	158,220.1
I-4	31,094.69	0.1888	138,800.1	156,621.5
I-5	28,393.75	0.1818	156,051.9	162,385.7
I-6	29,472.58	0.1802	148,284.2	152,085.8
I-7	19,674.26	0.1759	144,477.2	158,718.5

**Table 2:** Table of information related to 55 secondary nodes.

Node Number	Approaching Node	Freight Volume/t	Position Coordinate		Node Number	Approaching Node	Freight Volume/t	Position Coordinate	
			X	Y				X	Y
II-1	811	500.95	143,105.67	152,346.13	II-29	859	237.997	139,712.38	158,196.62
II-2	811	499.3	143,005.54	152,357.19	II-30	869	231.447	137,403.37	160,390.60
II-3	819	478.205	142,735.81	153,102.05	II-31	868	278.97	139,355.42	160,403.74
II-4	819	412.385	142,734.58	153,100.82	II-32	869	227.177	145,960.34	157,813.24
II-5	819	392.17	142,733.35	153,099.59	II-33	845	226.562	139,062.69	155,216.00
II-6	819	337.825	142,730.59	153,096.83	II-34	859	211.112	139,712.38	158,196.62
II-7	819	336.925	142,727.83	153,094.07	II-35	887	204.432	155,779.18	161,153.63
II-8	817	328.8	142,147.44	152,788.09	II-36	887	204.202	155,972.26	161,519.38
II-9	817	311.925	143,839.80	153,020.83	II-37	894	202.802	158,485.91	164,411.95
II-10	807	299.115	142,199.94	151,897.77	II-38	894	196.537	154,679.32	164,648.90
II-11	817	286.42	142,733.35	153,099.59	II-39	886	194.062	154,828.66	160,253.62
II-12	811	279.64	143,106.67	152,356.13	II-40	894	191.002	158,429.82	162,320.97
II-13	811	272.69	143,146.67	151,356.13	II-41	886	182.712	156,649.10	160,401.30
II-14	899	265.59	165,295.96	166,307.48	II-42	894	173.282	155,475.94	162,649.70
II-15	899	262.93	157,354.70	166,956.66	II-43	826	173.012	136,654.80	157,552.12
II-16	899	258.065	163,428.02	166,293.35	II-44	826	171.227	139,712.38	158,196.62
II-17	872	256.29	145,961.77	157,814.67	II-45	805	168.927	142,526.33	158,311.54
II-18	872	243.59	147,221.06	157,904.41	II-46	826	167.215	144,511.24	158,818.60
II-19	872	263.135	149,007.88	158,255.23	II-47	826	165.815	135,605.67	159,147.49
II-20	872	250.325	147,900.64	158,283.67	II-48	805	159.55	142,989.12	159,395.66
II-21	872	237.63	150,211.94	158,547.64	II-49	822	157.075	144,347.43	159,827.39
II-22	872	230.85	145,488.59	158,833.25	II-50	826	154.015	143,383.79	160,462.05
II-23	872	223.9	148,375.72	159,294.26	II-51	856	145.725	141,628.28	160,162.22
II-24	872	216.8	149,100.78	159,241.67	II-52	864	279.56	140,521.56	160,395.32
II-25	872	245.78	140,522.99	160,396.75	II-53	864	338.67	137,403.37	160,390.60
II-26	848	345.47	138,792.09	155,636.76	II-54	864	217.89	139,355.42	160,403.74
II-27	848	217.65	139,841.39	155,777.27	II-55	856	457.32	145,960.34	157,813.24
II-28	857	407.68	137,888.25	157,780.23					

**Table 3:** Scope of services for primary nodes.

Primary Node	Contains Secondary Nodes	Includes Service Area Centers
I-1	II-1~II-13	793, 795, 796, 797, 798, 800, 801, 802, 804, 806, 807, 809, 810, 811, 813, 814, 815, 816, 817, 818, 819, 820, 821, 823, 827, 828, 830, 833
I-2	II-14~II-16	892, 896, 897, 899, 900
I-3	II-17~II25	832, 836, 837, 838, 839, 840, 871, 872, 873, 874, 876, 877, 879, 880, 882, 884
I-4	II-25~II-32	841, 842, 843, 844, 845, 846, 847, 848, 849, 850, 851 852, 853, 854, 857, 858, 859, 862, 867, 868, 869
I-5	II-33~II-40	885, 886, 887, 888, 889, 890, 891, 893, 894, 895, 898
I-6	II-41~II-48	791, 792, 794, 799, 803, 805, 808, 812, 822, 824, 825, 826, 829, 831
I-7	II-48~II-55	834, 835, 855, 856, 860, 861, 863, 864, 865, 866, 870, 875, 878, 881, 883

## Data Availability

Data are contained within the article.
